# Quantitative Analysis of Gas Phase IR Spectra Based on Extreme Learning Machine Regression Model

**DOI:** 10.3390/s19245535

**Published:** 2019-12-14

**Authors:** Tinghui Ouyang, Chongwu Wang, Zhangjun Yu, Robert Stach, Boris Mizaikoff, Bo Liedberg, Guang-Bin Huang, Qi-Jie Wang

**Affiliations:** 1 Department of Electrical and Electronic Engineering, Nanyang Technological University, Singapore 639798, Singapore; cwwang@ntu.edu.sg (C.W.); N1806034K@e.ntu.edu.sg (Z.Y.); bliedberg@ntu.edu.sg (B.L.); EGBHuang@ntu.edu.sg (G.-B.H.); 2 Key Laboratory of In-fiber Integrated Optics, Ministry Education of China, Harbin Engineering University, Harbin 150001, China; 3 Institute of Analytical and Bioanalytical Chemistry, Ulm University, 89081 Ulm, Germany; robert.stach@uni-ulm.de (R.S.); boris.mizaikoff@uni-ulm.de (B.M.)

**Keywords:** gas sensing, quantitative spectrum analysis, feature selection, ELM-AE

## Abstract

Advanced chemometric analysis is required for rapid and reliable determination of physical and/or chemical components in complex gas mixtures. Based on infrared (IR) spectroscopic/sensing techniques, we propose an advanced regression model based on the extreme learning machine (ELM) algorithm for quantitative chemometric analysis. The proposed model makes two contributions to the field of advanced chemometrics. First, an ELM-based autoencoder (AE) was developed for reducing the dimensionality of spectral signals and learning important features for regression. Second, the fast regression ability of ELM architecture was directly used for constructing the regression model. In this contribution, nitrogen oxide mixtures (i.e., N_2_O/NO_2_/NO) found in vehicle exhaust were selected as a relevant example of a real-world gas mixture. Both simulated data and experimental data acquired using Fourier transform infrared spectroscopy (FTIR) were analyzed by the proposed chemometrics model. By comparing the numerical results with those obtained using conventional principle components regression (PCR) and partial least square regression (PLSR) models, the proposed model was verified to offer superior robustness and performance in quantitative IR spectral analysis.

## 1. Introduction

With the development of advanced technologies in medical, industrial, and environmental applications, gas sensing has been applied to play an essential role in many areas [1,2]. Currently, researches on gas sensing can mainly be divided as two parts: qualitative analysis and quantitative analysis [3]. Compared to aiming at only recognizing the components of a gas mixture, the latter obtains the concentration of gas components, which is relevant for industrial measurements, e.g., in the manufacturing industry, as well as transportation, environmental, and food security.

Quantitative gas analysis could benefit from a variety of technologies [4,5], among which gas chromatography (GC) and spectroscopic sensing are two frequently applied methods [6]. Gas chromatography is time consuming and operates discontinuously, whereas spectroscopic methods stand out due to their rapid response, compactness, and accuracy [7]. Moreover, spectroscopic methods can identify gases according to their more or less pronounced spectral signatures across the entire electromagnetic spectrum, and especially in near-infrared (NIR), mid-infrared (MIR), and Raman spectroscopy, which are commonly used for real time and in-field gas sensing applications [8,9,10,11]. Because of the intrinsic molecular selectivity, high sensitivity, and rapid response [12], spectroscopic gas sensing (usually based on recording emission or absorption spectra) are attractive for quantitative spectral analysis even in complex mixtures.

The principle of quantitative analysis in infrared spectroscopic gas sensing is based on the relationship between the magnitude of the absorptions in the spectrum and the gas concentration [13]. Regression models are built based on statistics or machine learning algorithms to quantitatively describe this relationship and to derive gas concentrations from that. The simplest models are so-called single-linear-regression (SLR) and multiple-linear-regression (MLR) models that utilize the intensity of absorption lines as input for the regression. These models have been successfully applied for, e.g., determining the total amount of carbon in unknown soil samples [14]. MLR was also used to analyse the Pb content of navel oranges [15], and to measure the quantity of trace metals in infant formula premixes [16]. Modern spectral analysis methods aim at extracting more in-depth features from spectra, and then construct a so-called multivariate regression model considering two key issues. The first one relates to the extraction of useful features from a complex pattern of spectral lines; the other concerns the construction of an effective regression model. For the first issue, generic methods are based on principle components analysis (PCA), which aims at extracting the most important features from raw data while reducing the dimensionality at the same time [17]. Principle components regression (PCR) and partial least square regression (PLSR) are the two most commonly used models in multivariate quantitative spectral analysis [18,19]. For instance, PCR and PLSR were used to predict the protein content and hardness value of Canadian wheat [20]. They were also reported to extract multi-elemental concentrations from stainless-steel samples using laser-induced-breakdown spectroscopy [21]. In PCR and PLSR models, the regression analysis is realized via simple linear regression. With the advancement of machine learning algorithms, the combination of PCA with these techniques enables the development of advanced regression models. For example, a support vector regression model (SVR) combined with PCA for the quantitative elemental analysis of solid samples based on microwave plasma atomic emission spectrometry [22,23].

In this paper, we propose an advanced regression model to enhance the performance with respect to the linear regression methods mentioned above. Contributions of this paper are concluded as the following: (i) A fast and advanced machine learning model is proposed for IR spectra analysis in this paper. The proposed model is based on the so-called extreme learning machine algorithms (ELM) [24,25], which is based on a single-hidden-layer-feedback-network. Compared with other machine learning algorithms, ELM offers fast learning speeds, and excellent feature learning abilities. It is also ideal for handling large data sets, such as high-resolution spectral data; (ii) An advanced feature learning method is proposed. A new ELM architecture is applied in the proposed model for dimension reduction and feature learning, namely ELM-based auto-encoder (ELM-AE) [26]. Compared with PCA methods, ELM-AE was able to learn linear and nonlinear features with fast random projection. By setting the direction to fit the objective, the newly learned features could then enhance the regression ability; (iii) Fast and direct regression modelling method is applied in this paper. Considering parameters of the conventional ELMs are trained based on least square methods, ELM-AE also provided superior linear learning abilities. Therefore, combined with features learned from ELM-AE, the ELM architecture could be directly utilized for regression analysis in gas sensing. Based on the proposed method, both simulated data and experimental data were studied for validating the performance quantitative gas mixture analysis.

## 2. Background Knowledge

According to the above description, the work determining the concentration of individual gas components in mixtures mainly involves with quantitative analysis in IR gas spectra. The methodology used herein relies on three main parts of background knowledge: (i) data pre-processing, (ii) predictive regression modelling, and (iii) performance evaluation. Some general work on these parts are presented as below.

### 2.1. Data Pre-Processing

Data pre-processing of complex IR spectra not only aims at extracting useful information discriminatory from interferences, e.g., data de-noising, normalization, and feature selection, but also specific processes such as baseline correction, optimizing the input spectral range, etc. [27]. In regression analysis, data pre-processing is expected to generate a reliable database for constructing a precise and robust relationship between input and output. Therefore, feature selection or variable selections is highly relevant. It is known that generic IR gas spectra may be composed of thousands of emission/absorption lines (i.e., variables), especially if high-resolution data is recorded [28]. However, it is detrimental to use all available variables for modelling, as a large number of variables not only increases the complexity and computation time, but also considers noise. Therefore, suitable variable selection is relevant to filter out noise, and build models using only variables carrying essential analytical information. A commonly-applied method is based on the selection of the suitable wavelength regimes, while avoiding spectral segments that do not provide molecularly relevant signatures, thereby also reducing computational expense. In addition, dimension reduction algorithms are usually applied, such as PCA which could be realized by Karhunen–Loeve transform (KLT) [29,30] generating orthogonal and independent feature vectors of the original data. By taking eigenvectors with the former largest eigenvalues to construct a transform matrix, the important features (then called ‘principal components’) [19] are selected with dimensionality reduction and having great explanation ability to the original data matrix. Thus, PCA is widely used for dimensional reduction and for de-noising.

### 2.2. Regression Analysis

Based on the pre-processed data, one may then focus on establishing predictive models for qualitative analysis (i.e., classification) and/or quantitative analysis (i.e., regression) of unknown samples. Since the prediction of the component concentration (i.e., quantitative analysis) is the focus of the present study, only regression modelling is considered in the following. Given the wide variety of applicable regression models including SLR, MLR, PCR, PLSR, SVR, NN, etc. [18], PCR and PLSR are considered among the most useful ones for the analysis of IR gas phase spectra. 

#### 2.2.1. PCR

PCR is a linear regression model based on PCA [31]. Compared with SLR/MLR using the most important variables in the original feature matrix directly, PCR regresses the target based on the principal components of the feature matrix, namely, transformed features, which may then be used to reproduce the original data. The expression of a PCR model is as follows:

(1) $$X=S_{X}\cdot F_{X}+E_{X}$$

(2) $$Y=S_{X}\cdot C+E$$

whereby Equation (1) is the expression of PCA decomposition; ***X*** is the original spectral matrix; and ***F_X_*** and ***S_X_*** represent the loadings and score matrix of ***X***, respectively. Equation (2) is the final regression model, where ***Y*** represents the concentration matrix in gas mixtures; ***C*** is the regression coefficient matrix; and ***E_X_*** and ***E*** represent residual errors in the two equations.

#### 2.2.2. PLSR

PLSR model is another useful model for the quantitative analysis of complex spectra. Different from PCR, PLSR extracts latent variables of both the original spectrum X and the target Y, and then constructs the regression model between latent variables. The PLS models are implemented as follows:

(1) Extracting latent variables

(3) $$Y=S_{Y}\cdot F_{Y}+E_{Y}$$

The latent variables of ***X*** are still extracted via Equation (1), and those of ***Y*** are extracted via Equation (3) whereby ***F_Y_*** and ***S_Y_*** represent the loadings and score matrix respectively, and ***E_Y_*** is the residual error matrix.

(2) Modelling the regression relationship

Assuming that these two latent variable matrices (SX and SY) are correlated to each other, then one may construct a regression model describing this relationship as:

(4) $$S_{Y}=S_{X}\cdot C+E$$

whereby ***C*** is the matrix reflecting the regression coefficients between ***S_X_*** and ***S_Y_***, and ***E*** is the residual error. From the description above, it is evident that the aim of PLS modelling is to decompose both ***X*** and ***Y*** into two loadings and scores matrices, and build a regression model between the score matrices of ***X*** and ***Y*** with maximum covariance.

Based on the PCR and PLSR described in Equations (2)–(4), it is evident that these two models are linear in describing the relationship between concentration and spectral signals, and that by using the obtained regression coefficients one may predict the concentration of a gas component of interest within an unknown sample.

### 2.3. Evaluation Metrics

Next to establishing a useful model, it is essential evaluating the performance of regression models via appropriate evaluation metrics. A wide variety of metrics are defined for regression and prediction analysis [27], e.g., regression error metrics like root mean square error (RMSE), mean absolute percentage error (MAPE), mean absolute error (MAE), and other correlation metrics like the coefficient of determination values (*R*^2^). Considering that conventional error metrics are generally correlated, they are all expected to be close to 0 for well-performing regression models. *R*^2^ describes how the variance of the dependent variable is influenced by the independent variable(s). Hence, the independent variables are regarded as significantly important when the value of *R*^2^ is close to 1. Therefore, in this study two typical metrics were selected, namely RMSE and *R*^2^ for evaluating performance of quantitative analysis.

(5) $$RMSE=\sqrt{\frac{\sum_{i=1}^{n}{\left(y_{i}-{\hat{y}}_{i}\right)}^{2}}{n}}$$

(6) $$R^{2}=1-\frac{SS_{res}}{SS_{tot}},\;\left\{ \begin{array}{l} SS_{res}=\sum_{i=1}^{n}{\left(y_{i}-{\hat{y}}_{i}\right)}^{2}\\ SS_{tot}=\sum_{i=1}^{n}{\left(y_{i}-{\bar{y}}_{i}\right)}^{2} \end{array}\right.$$

whereby *y_i_* and ${\hat{y}}_{i}$ represent the *i*th measured and predicted concentration of the given gas component, and *n* is the number of gas samples.

## 3. ELM-AE-Based Regression Model (ELM-AE-R)

In this study, we propose an advanced regression model based on ELM and ELM-AE, as described in detail below. 

### 3.1. ELM Architecture

ELM was developed by Huang et al. [32] based on the architecture of single hidden layer feed forward networks (SLFNs). This novel machine learning algorithm has been successfully employed in a wide variety of fields, e.g., feature learning, dimension reduction, classification, and regression. Compared with conventional neural networks, its success mainly results from the following three aspects.

(1) With randomly generated weights in the input layer, ELM shows excellent generalization performance, and lends itself to real-world application scenarios.
(2) Compared with conventional neural networks whose parameters, e.g., learning rate, learning epochs, and local minima are tuned iteratively, ELM fixes the input weights to obtain extremely fast learning speed.
(3) ELM can be easily implemented to achieve both the smallest training error and the smallest norm of weights.

According to the topological structure of SLFNs, a generic ELM network can be constructed. Assuming there are *N* data samples (***x****_i_*, *t_i_*), where ***x****_i_* = [*x_i_*_,1_, *x_i_*_,2_, …, *x_i,c_*] ∈ ***R****^c^* is the input vector and *t_i_* is the target, then the ELM network with *L* hidden nodes can be modelled as follows:

(7) $$o_{i}=\sum_{k=1}^{L}\beta_{k}g_{k}(x_{i})=\sum_{k=1}^{L}\beta_{k}g(x_{i}\cdot w_{k}^{}+b_{k});\;i=1,2,\cdots N;$$

where, ***W*** = [***w***_1_, ***w***_2_, …, ***w****_L_*] is the weight matrix between input layer and hidden layer; ***b*** = [*b*_1_*, b*_2_*, …, b_L_*] is the bias vector; *g*(*) is the active function which could be linear or nonlinear; ***β*** = [*β*_1_, *β*_2_, *…*, *β_L_*]*^T^* is the output weight matrix; *o_i_* is the *i*th ELM output. By transforming the above formula into matrix form, Equation (7) is rewritten as below.

(8) O=Hβ

where, ***O*** = [*o*_1_, *o*_2_, …, *o_N_*]*^T^* is the final output matrix; ***H*** is the hidden layer output matrix, expressed as

(9) $$H={\left[\begin{array}{ccc} g(x_{1}\cdot w_{1}^{}+b_{1}) & \cdots & g(x_{1}\cdot w_{L}^{}+b_{L})\\ \vdots & \ddots & \vdots \\ g(x_{N}\cdot w_{1}^{}+b_{1}) & \cdots & g(x_{N}\cdot w_{L}^{}+b_{L}) \end{array}\right]}_{N\times L}$$

To train the optimal ELM network, we assume the objective is to minimize the error between model outputs and targets, expressed as

(10) $$Minimize\;{\left\|O-T\right\|}^{2}=\sum_{i=1}^{N}{\left\|o_{i}-t_{i}\right\|}^{2}$$

where, ***T*** = [*t*_1_, *t*_2_, *…, t_N_*]^T^ is the target matrix. By plugging Equation (8) into the objective function Equation (10) and adopting the least square method for solution, the output weights ***β*** can be calculated as the follows:

(11) $$\underset{\beta }{\min}\;{\left\|H\beta -T\right\|}^{2}\to \beta =H^{†}T$$

where $H^{†}$ is the Moore-Penrose generalized inverse of the hidden layer output ***H***, that can be calculated as $H^{†}$ = (***H****^T^**H***)^−1^***H****^T^*.

### 3.2. ELM-AE

**Figure 1:**
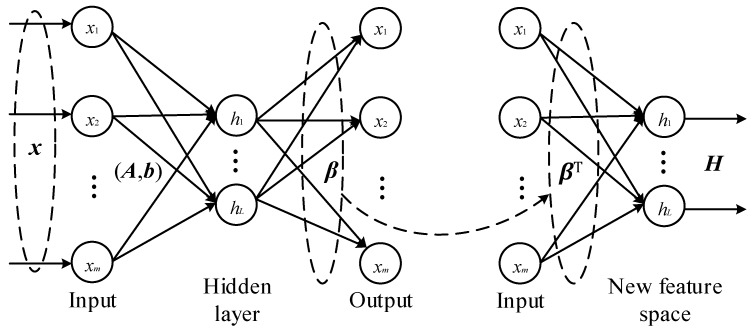
Architecture of the proposed extreme learning machine-based auto-encoder (ELM-AE).

Based on the description of modelling ELM networks, if we set the target ***T*** = ***X***, then ELM becomes a self-learning network as auto-encoder (AE) [33], which is called ELM-based auto-encoder (ELM-AE). Conventional AEs are formed by a pair of encoder and decoder: the encoder for new features learning, and the decoder for feature reconstruction, such that the new ELM-AE can be constructed as shown in Figure 1.

It is evident from Figure 1 that there is a single hidden layer in ELM-AE, which has randomly generated weights and biases for encoding. Therefore, the hidden outputs (encoder outputs) of a given data ***x*** can be expressed as

(12) $$h\left(x\right)=\left[h_{1}\left(x\right),\cdots ,h_{L}\left(x\right)\right]=\left[g\left(\langle a_{1},x\rangle +b_{1}\right),\cdots ,g\left(\langle a_{L},x\rangle +b_{L}\right)\right]$$

To improve the generalization performance of ELM-AE, these randomly generated parameters ***A*** and ***b*** are usually chosen to be orthogonal,

(13) $$\left\{ \begin{array}{l} A^{T}A=I\\ b^{T}b=1 \end{array}\right.$$

Via these orthogonal random parameters, the Euclidean information of input data is retained by ELM-AE, as described in Johnson–Lindenstrauss Lemma [34]. 

Then, as the description of AE, one can re-represent the original feature space through the decoders of ELM-AE. As ELM is a universal approximator, the output layer (decoder) of ELM-AE can be utilized to approximate any given function. According to the description above, the objective of ELM-AE decoder is to retain the information of input features as more as possible, i.e., approximating the original input, namely ***T*** = ***X***. Therefore, the objective function in (11) can be expressed as:

(14) $$\underset{\beta_{AE}}{Minimize}:\;{\left|\left|H\beta_{AE}-X\right|\right|}^{2}$$

where, ***β***_AE_ is the output weights; ***H*** is the hidden layer matrix consisting of ***h***(***x***) in ELM-AE. According to the assumption of zero bias (*b_i_* = 0), the output weights ***β***_AE_ could be simply calculated through (11). Then, the new architecture of ELM-AE is constructed based on the randomly generated parameters (***A***, ***b***) and the optimal output parameters ***β***_AE_.

While, considering that the purpose of AE is to learn features as described above, one may utilize the optimal output weights ***β***_AE_ to construct a new network for feature representation, as shown in Figure 1. The final representation of the original data is then expressed as

***X**_new_* = ***Xβ**^T^*_AE_ (15)

where, ***X****_new_* represents the newly learned features which can replace the original data for future analysis. 

On the other hand, by setting different values of *L*, we can see from (15) that ELM-AE can project the input data into a higher (*L > m*), equal (*L* = *m*) or lower (*L* < *m*) dimension space of ***X****_new_*. Especially, if *L* < *m*, ELM-AE also can be utilized for dimension reduction analysis such as PCA. 

### 3.3. ELM-AE-R for Quantitative Analysis of IR Spectra

**Figure 2:**
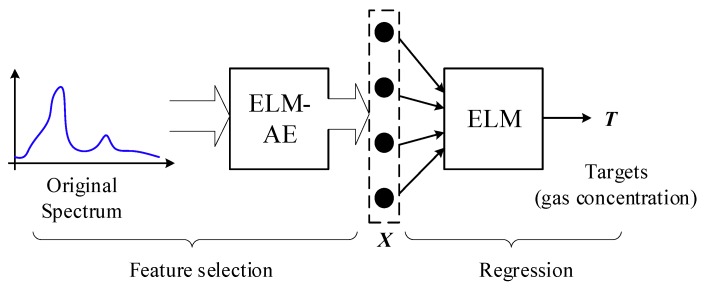
Framework of the proposed model for quantitative IR spectra analysis.

According to the description above, by capitalizing on the advantages of ELM-AE and its pronounced feature learning and dimension reduction ability, one can also resemble the utility of PCA in spectroscopic gas sensing. As the ELM architecture offers fast computation speeds for large data sets, as well as linear and nonlinear learning abilities, we propose herein a new ELM-based model for quantitative IR spectra analysis, as shown in Figure 2. 

In Figure 2 it is illustrated that the framework of the proposed quantitative analysis contains two parts: feature selection and regression. In the first part, we propose to utilize ELM-AE for feature selection given the complexity of IR spectra across a broad wavelength regime, especially in high-resolution laser spectroscopies. This situation requires dimensionality reduction for input data facilitated by a feature selection process. Compared with conventional spectra analysis using PCA for dimension reduction, the proposed ELM-AE can not only realize dimension reduction while satisfying *L* < *m*, but simultaneously learns features within the original data matrix. Furthermore, for achieving high performance at calculating the concentration of gas components, some modifications of the generic ELM-AE are considered in this study. One modification targets the selection of the active function in hidden layers of ELM-AE. PCR and PLSR both perform well in a linear data space. Therefore, it is worthwhile also in ELM-AE using linear functions for the active function *g*(*). The other modification concerns the input parameters ***A***. Different from generic ELM-AE using randomly generated parameters, during the present study the parameter matrix ***A*** was generated in a supervised way following:

(16) $$\begin{array}{l} A=Orthogonalize(P_{ara});\\ \left\{ \begin{array}{l} P_{ara}=[X^{†}Y,\;R_{m}];\\ A^{T}A=I \end{array}\right. \end{array}$$

where ***X*** and ***Y*** are the original spectral data and concentration matrix, $X^{†}Y$ reflects the correlation between input and output, and ***R****_m_* is randomly generated matrix. By initializing the parameter matrix ***A*** via (16), the finally learned features in ELM-AE offer substantial self-learning abilities in PCR, and target-learning abilities in PLSR. Finally, as described in Figure 2 the second part was to realize the regression analysis. Considering that ELM may equally well perform regressions, the ELM architecture was directly adapted also to regression analysis.

## 4. Experiments

### 4.1. Generation of Simulated Data

To study the performance of the proposed approach in calculating gas concentrations, in a first step simulated spectral datasets were used. In this study, three gas components—N_2_O, NO_2_ and NO—were targeted for quantitative analysis. To obtain simulated datasets, pure gas spectra were calculated based on the HITRAN Database [35]. Then, a simulated spectrum of a mixture of gases was generated by adding pure spectra of the constituents with different multiplication factors. 

**Figure 3:**
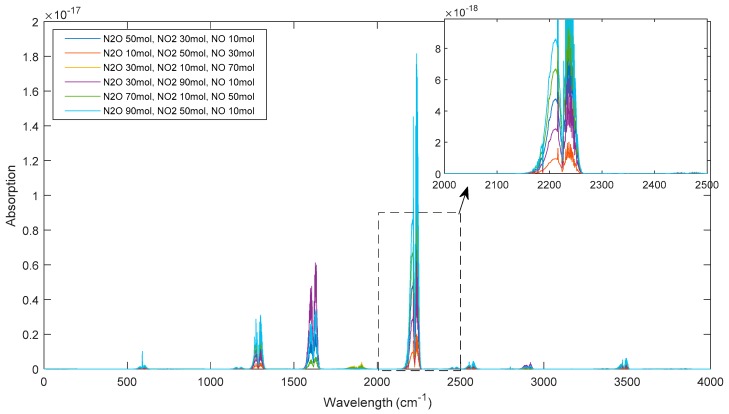
Simulated spectra of six selected mixture samples.

Considering the standard spectra in HITRAN are calculated per mol, the concentration of gas components in the simulated datasets are also expressed by the number of molecules. By assuming the wavelength range 0–4000 cm^−1^ as the spectral range of interest at a spectral resolution of 1 cm^−1^, 60 simulated mixture sample spectra of N_2_O/NO_2_/NO were generated serving as the training dataset. In order to make these training samples discriminative, the concentration of three components were set in the range from 10 mol to 90 mol in increments of 20 mol; all three components had therefore different concentrations in any given mixture sample (Table 1 and Figure 3). 

Table 1 summarizes the concentration of gas components N_2_O/NO_2_/NO in the training mixture samples, while Figure 3 shows selected simulated spectra (i.e., six selected examples) from the training dataset. 

### 4.2. Analysis on Simulated Data

To calculate the concentration of the gas components, one needs to build regression models. Here, three models (PCR, PLSR, and the proposed ELM-AE-R) were compared. First, the simulated datasets were considered for evaluating the feature selection process along with dimensionality reduction prior to the regression analysis.

**Figure 4:**
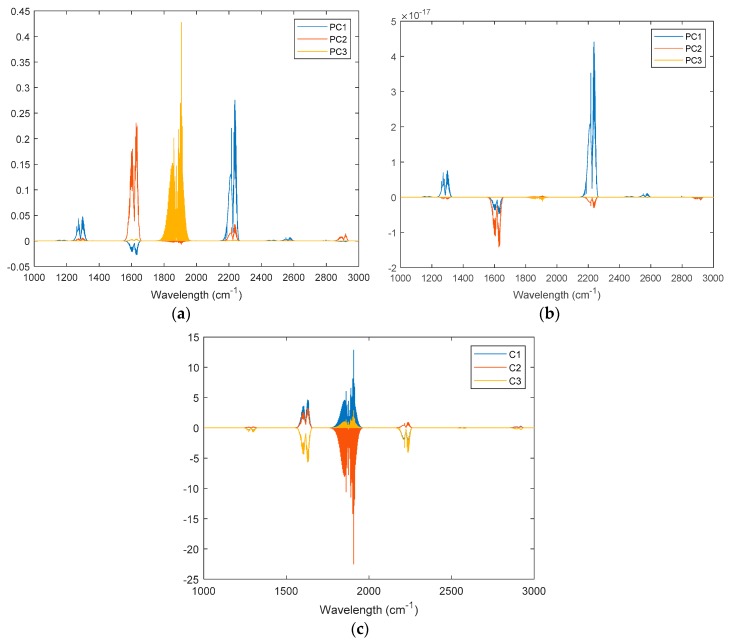
Feature loadings in the three investigated models; (**a**) principle components regression (PCR); (**b**) partial least square regression (PLSR); (**c**) ELM-AE-based regression (ELM-AE-R). Here, PC*_i_* means the *i*th most important principle component, and *C_i_* means the *i*th feature component learned by ELM-AE.

Figure 4 shows the feature loadings of the three investigated regression models. For PCR and PLSR, the loadings were the principle components. For ELM-AE-R, the loadings were the learned feature vectors. Based on these feature loadings, latent variables of spectral signals could be calculated. Then, three regression models were constructed according to the description in Section 2.3. 

**Figure 5:**
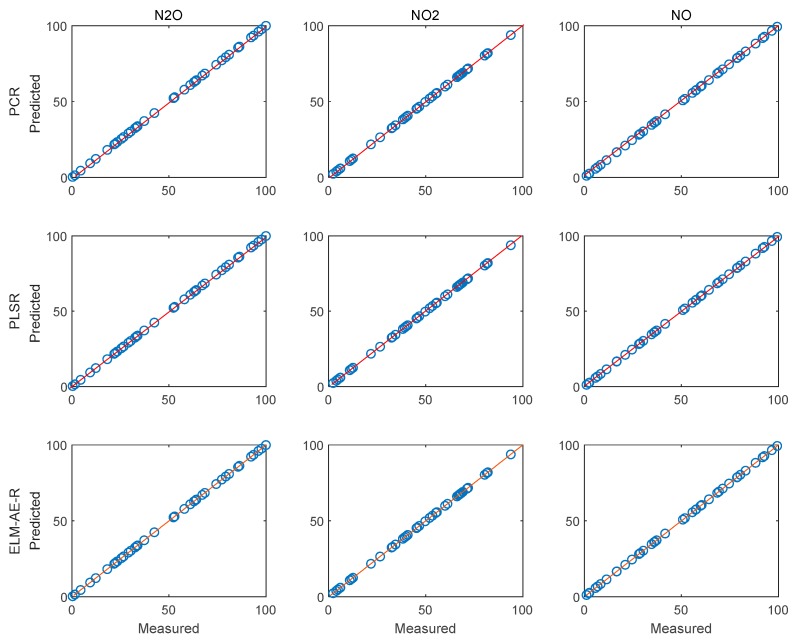
Results of the regression analysis for simulated quasi unknown spectra.

To discuss the performance of the constructed models, 40 samples of NO/NO_2_/N_2_O mixtures with random concentrations were separately generated based on standard spectra in HITRAN. The results of the regression analysis on predicting the concentrations of the gas components in these quasi unknown samples are shown in Figure 5. It is immediately evident that the data points fall on the red line indicating ideal prediction. These ideal results are expected, as simulated data are free from noise or interferences. Consequently, the performance of the three models was also identical.

### 4.3. Actual Spectra Collection and Processing

To collect real spectra, Fourier transform infrared (FTIR) spectroscopy was used in combination with substrate-integrated hollow waveguide (iHWG) technology simultaneously serving as highly efficient gas cell [36,37,38]. Compared with other sensor technologies such as electrochemical and semi-conductor-based devices, IR spectroscopy/sensing enables monitoring multiple gas components even in complex mixtures. In essence, IR techniques operating in the 3–15 µm (i.e., mid-infrared) wavelength band are capable of distinguishing polyatomic and hetero-nuclear diatomic molecules providing a unique “fingerprint” for each component within mixture IR spectra [39], as shown herein for the absorption spectra of mixtures of N_2_O/NO_2_/NO.

**Figure 6:**
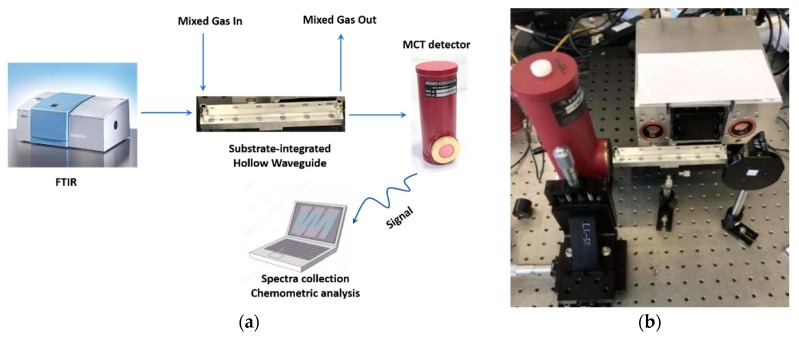
IR gas sensing system. (**a**) schematic; (**b**) physical device: 1) HgCdTe detector (FTIR-16−2.00 MSL-12, InfraRed Associates Inc., Stuart, FL, USA; kept at 77 K via liquid nitrogen; 2) iHWGs (fabricated from either brass or aluminum. The assembled iHWGs had dimensions of 250 × 25 × 20 mm3 or 150 × 25 × 20 mm3 (L × W × H); 3) Compact FT-IR spectrometer (Alpha OEM, Bruker Optics Inc., Ettlingen, Germany).

**Figure 7:**
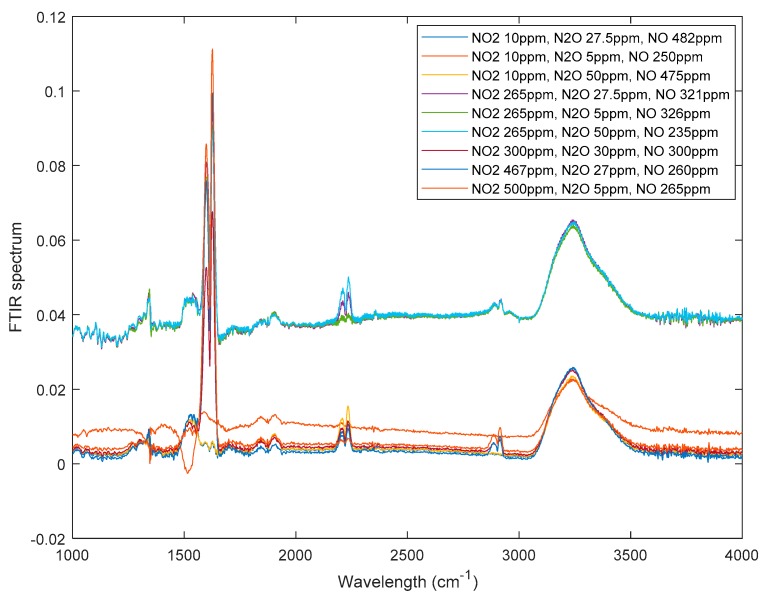
Examples of experimentally collected IR spectra.

Using the IR sensing configuration shown in Figure 6, spectral data of 356 N_2_O/NO2/NO mixtures were collected across a wide variety of concentrations. Figure 7 shows selected exemplary spectra.

**Figure 8:**
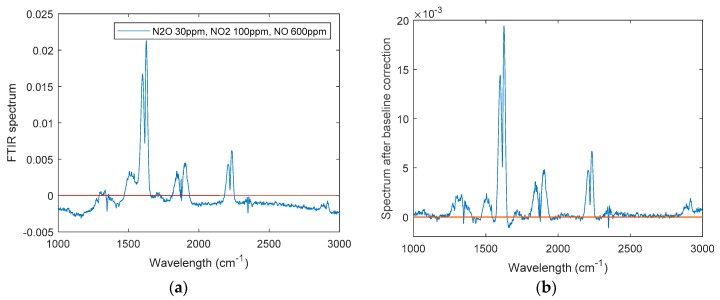
Baseline correction shown for an exemplary IR spectrum recorded during the present study. (**a**) original spectrum; (**b**) spectrum after baseline correction.

The collected wavelength range was 1000–4000 cm^−1^. It is evident from Figure 7 that the raw IR spectra are affected by several parameters including, e.g., baseline drifts, background signals, noise, molecular interferants such as CO_2_, etc. Therefore, data pre-processing is required to obtain useful input data for the regression analysis including baseline correction. In this study, asymmetric least squares smoothing (ALS) was applied [40]. ALS aims at obtaining a smooth baseline, which follows the main baseline trend of the original spectrum. The objective function of ALS is defined as:

(17) $$S=\sum_{i=1}^{n}\alpha_{i}{\left(y_{i}-y_{b,i}\right)}^{2}+\lambda \sum_{i=1}^{n-1}[(y_{b,i+1}-y_{b,i})-(y_{b,i}-y_{b,i-1}){]}^{2}$$

where *y* is the original spectra signal; *y_b_* is the calculated baseline; *n* is the number of spectral elements; *α_i_* is the weight for the *i*th point in spectrum; and *λ* is a balance factor, whose values generally are set as 10^2^ < *λ* < 10^9^. By minimizing the objective function in Equation (17), one may extract a useful baseline for correction, as shown in Figure 8.

Figure 8 shows IR spectra of an exemplary dataset for a mixture of N_2_O (30 ppm), NO_2_ (100 ppm), and NO (600 ppm) before and after baseline correction. All collected spectra were then processed by ALS prior to the regression analysis. 

### 4.4. Regression Analysis and Concentration Prediction of Measurd Spectra

To construct a regression model analysing the concentration of gas components, the dataset was divided into a training dataset for modelling, and a test dataset for evaluation, i.e., 189 and 167 samples, respectively. Feature selection and dimension reduction were implemented before modelling to reduce computational cost. Again, the performance of PCR, PLSR, and the proposed ELM-AE-R were compared.

**Figure 9:**
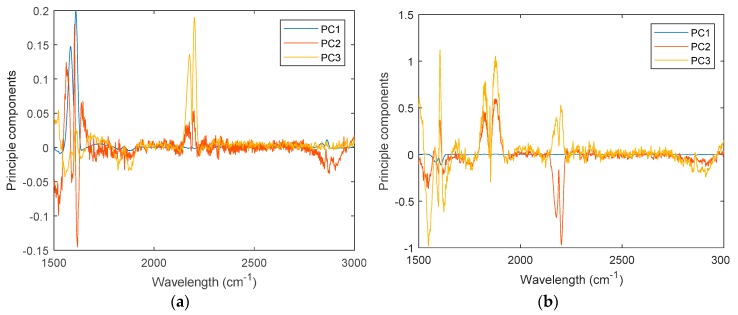

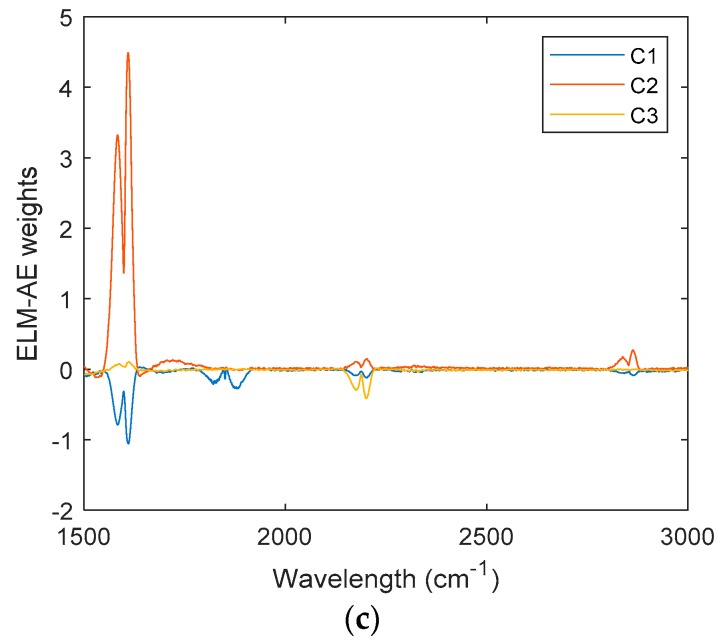
Feature loadings for the three models. (**a**) PCR; (**b**) PLSR; (**c**) ELM-AE-R.

**Figure 10:**
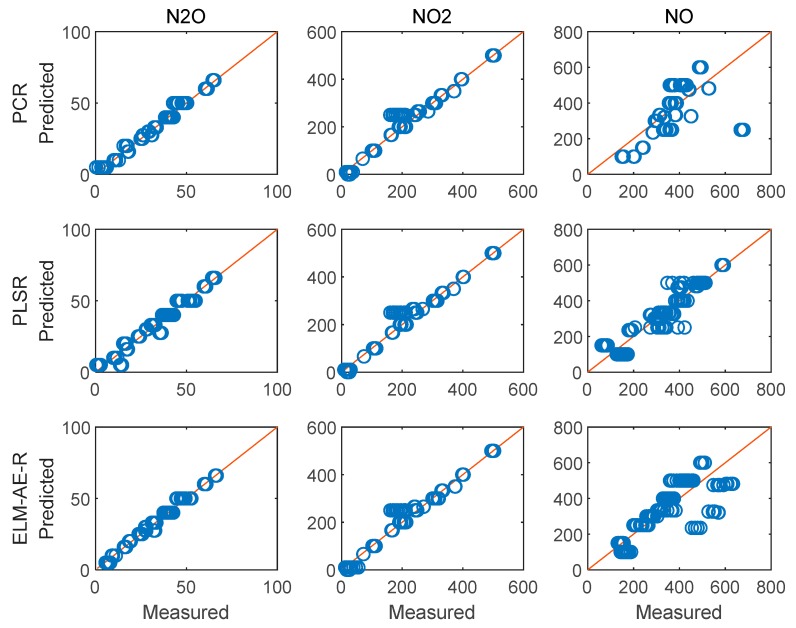
Performance of the regression analysis for the training data set.

**Figure 11:**
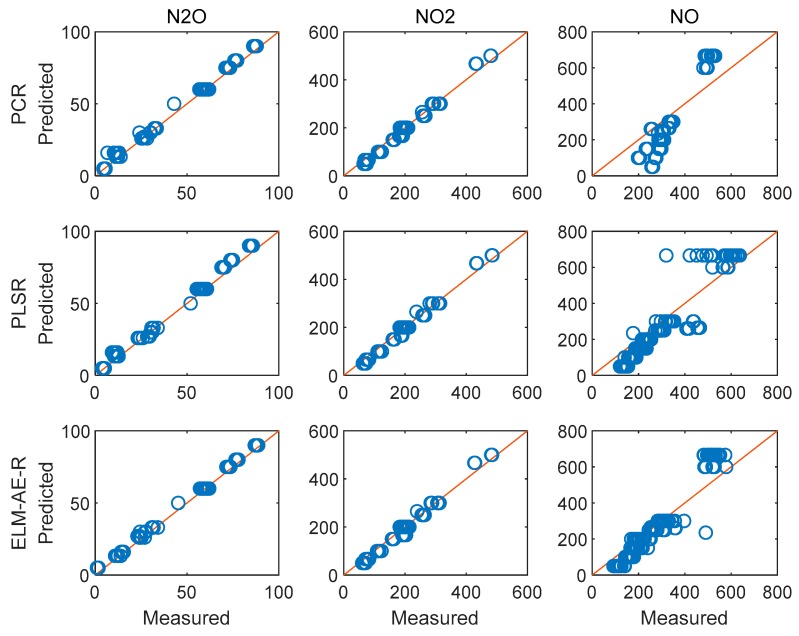
Performance of the regression analysis on the testing data set.

Figure 9 depicts the feature loadings of the three regression models. Three principle components were selected in PCR and PLSR, while the number of nodes in the hidden layer of ELM-AE was also set as three. Based on the feature loadings evident in Figure 9, latent variables for training data were calculated, and three regression models were established. The performance of predicting the concentration of the three gas components in the test data set is shown in Figure 10 and Figure 11.

Figure 10 and Figure 11 illustrate the performance of predicting concentrations for the training and the test dataset, respectively. The diagonal red line represents an ideal prediction; conversely, points located close to the diagonal line indicate better performance. It is evident that all models perform better on predicting the concentration of N_2_O and NO_2_ vs. NO. In order to quantitatively discuss the performance of these models, RMSE and R^2^ were calculated and summarized in Table 2.

From the results in Table 2, determining the best performing model is not immediately evident. To analyse the relative performance of the proposed ELM-AE-R, the improvement coefficient [41] was calculated as a percentage vs. PCR and PLSR serving as references, respectively. The improvement coefficient of RMSE is defined as:

(18) $$I=\frac{E_{ref}-E}{E_{ref}}\times 100\%$$

where *I* represents the improvement coefficient. For *I* > 0, the ELM-AE-R outperforms the reference model; if *I* < 0, the ELM-AE-R is worse than PCR or PLSR. For R^2^, the improvement coefficient could be determined by the difference vs. the reference model, which results in the degree of improvement of ELM-AE-R vs. PCR and PLSR as summarized in Table 3.

The results in Table 3 show that PLSR performs best on the training data, however, the proposed ELM-AE-R outperformed both PCR and PLSR on the test dataset, which corresponds to the real-world scenario of an unknown sample containing the three components. Moreover, the improvement coefficients of ELM-AE-R vs. PCR were larger than of ELM-AE-R vs. PLSR implying that ELM-AE-R performed best, while PLSR performed still better than PCR.

### 4.5. Improvement Analysis 

The regression analysis discussed in Section 4.4 is not perfect, as using only three principal components (PCs) may lead to a loss in information. In order to improve the performance, in a next step more principle components were extracted and the number of PCs used for modelling was optimized. 

**Figure 12:**
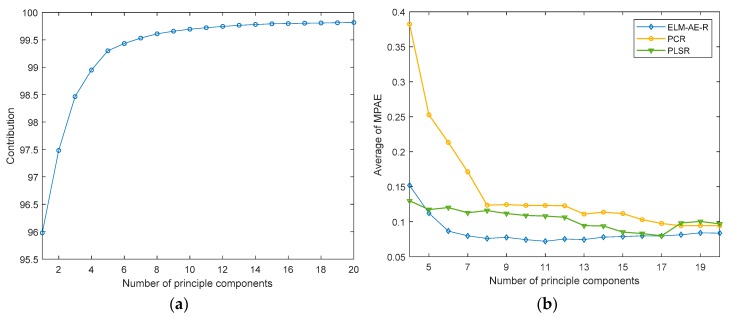
Contribution (**a**) and regression error (**b**) with an increasing number of principal components (PCs) used in the regression model.

**Figure 13:**
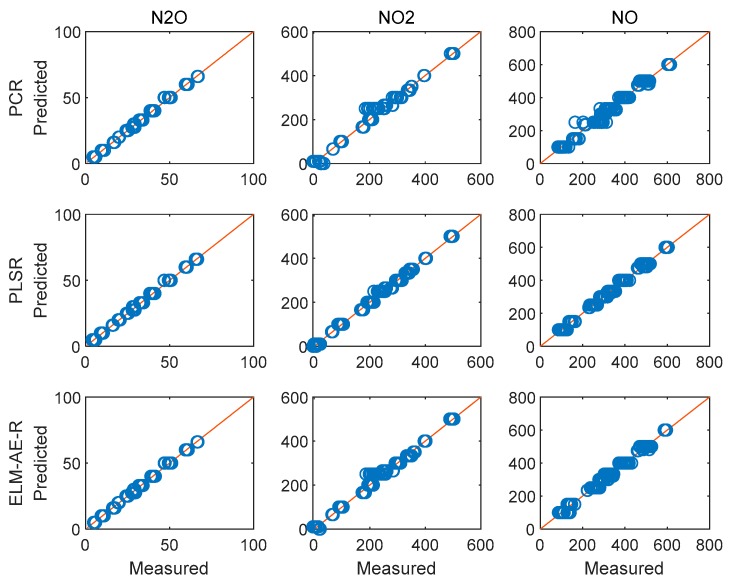
Performance of the regression analysis for the training dataset using 11 PCs.

**Figure 14:**
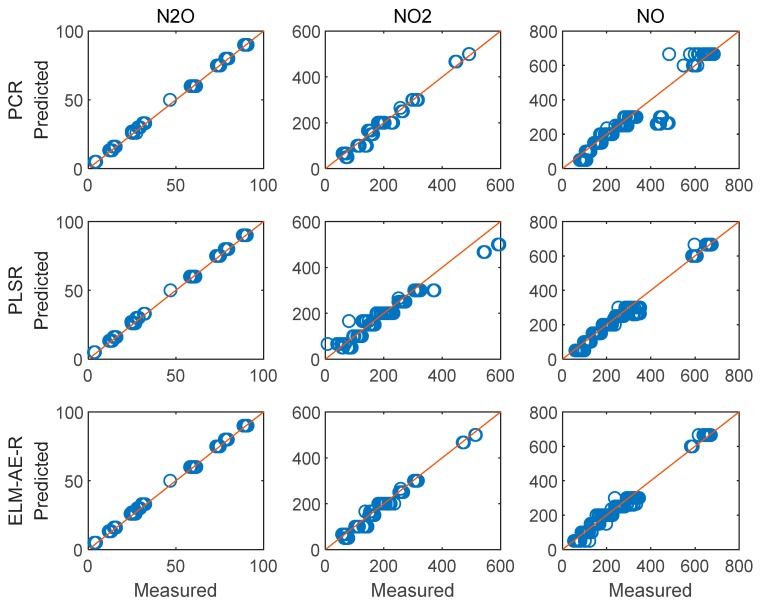
Performance of the regression analysis for the test dataset using 11 PCs.

In Figure 12a, the contribution of an increasing number of PCs in PCA is shown. In Figure 12b, the average regression error of the three models with increasing number of PCs is shown. While RMSEs of predicting different gas components have different magnitudes, the average of these RMSEs directly will hide the influence of good prediction models, e.g., that of N_2_O herein. Therefore, we propose to use MAPE to calculate the average regression error, which keeps the same variance trend as RMSE. According to results in Figure 12b, one may derive that predictive errors in PCR decreased with the number of PCs, yet remained constant beyond eight PCs. PLSR showed the best nominal performance if 17 PCs were selected. The proposed ELM-AE-R achieved the smallest regression error using around 11 PCs. It is again obvious that ELM-AE-R outperformed PCR and PLSR in most cases, and that PLSR outperformed PCR. Considering less PCs (not enough features) and more PCs (may introduce noise) were not suitable in modelling; using 11 PCs for modelling the target analyzes appeared most suitable. The corresponding results of the regression analysis are shown in Figure 13 and Figure 14.

Figure 13 and Figure 14 show the performance of the three models for predicting the concentration of the three gas components. Evidently, all models perform better on training data and test data vs. using only three PCs (cf. Figure 10 and Figure 11). The performance values are summarized in Table 4.

When using 11 PCs, PCR performed well when predicting the concentration of N_2_O. ELM-AE-R achieved excellent performance on the test dataset, while PLSR had advantages on the training process, yet, remained less robust vs. ELM-AE-R when evaluating test data. To comprehensively analyse the performance of the proposed ELM-AE-R model, averages of the relative improvement coefficients were again calculated by taking PCR and PLSR as reference respectively. On the training dataset, the average improvement coefficients of ELM-AE-R compared to PCR and PLSR on RMSE were 15.10% and −10.88%, and on R^2^ 0.39% and −0.02%. On the test dataset, ELM-AE-R outperformed PCR and PLSR at 21.16% and 17.45% on RMSE, respectively, and at 2.87% and 2.04% on R^2^. These results illustrated that the proposed ELM-AE-R indeed achieves a better overall performance vs. PCR and PLSR for quantitative IR spectral data analysis, and represents an excellent alternative vs. conventional multivariate data evaluation techniques in complex gas sensing scenarios. 

## 5. Conclusions

In this study, an innovative ELM-based regression model is proposed for the quantitative analysis of infrared spectra obtained via sensing gas mixtures. An ELM-based autoencoder has been applied for feature selection. Compared with conventional feature selection methods based on PCA, ELM-AE achieves both dimension reduction and simultaneous feature learning abilities. Then, by using the reduced features from ELM-AE, an ELM-based regression model was established and tested using simulated IR spectra as well as experimentally obtained data for a mixture of three gases —N_2_O, NO_2_, and NO, respectively. The proposed ELM-AE-R has demonstrated good comprehensive performance with particular benefit of improved robustness when predicting concentrations of the three target gas components. When compared with PCR using PCA for dimension reduction, both PLSR and the proposed ELM-AE-R learned dimensionality-reduced features via supervised learning toward to the target, so they can achieve better performance than PCR. On the other hand, ELM-AE-R have the learning ability of generating quantities of potential features, but PLSR cannot, therefore the proposed model could be robust to reach the best regression accuracy in all models.

However, besides the above contributions achieved in this paper, some potential issues are worth for studying. For example, how and where to apply the proposed model. Making use of gas sensing technologies could benefit a lot to our industries and society, e.g., applying the research in this paper for measurement of vehicle exhaust. Moreover, from the perspective of algorithms, how to improve model’s stability is also important, since random projection would be hidden bugs weakening the prediction performance. Therefore, more work could be executed in our following study.

## Figures and Tables

**Table 1:** Concentration of gas components in the training dataset.

	NO/mol	NO_2_/mol	N_2_O/mol
1	10	30	50
2	10	50	30
3	30	10	50
4	30	50	10
5	50	10	30
6	50	30	10
⋮	⋮	⋮	⋮

**Table 2:** Results of evaluation metrics during regression analysis (three PCs).

**Training Data**	**RMSE**			**R^2^**		
	**N_2_O**	**NO_2_**	**NO**	**N_2_O**	**NO_2_**	**NO**
PCR	2.2417	17.6933	99.5121	0.9716	0.9819	0.5335
PLSR	3.2259	18.0488	44.0656	0.9413	0.9812	0.9085
ELM-AE-R	1.7082	18.5939	89.4675	0.9835	0.9801	0.6229
**Testing Data**	**RMSE**			**R^2^**		
	**N_2_O**	**NO_2_**	**NO**	**N_2_O**	**NO_2_**	**NO**
PCR	2.2966	14.3814	121.2787	0.9900	0.9823	0.6129
PLSR	3.2336	14.3355	86.3352	0.9803	0.9824	0.8038
ELM-AE-R	1.8801	14.5293	74.5609	0.9933	0.9819	0.8537

**Table 3:** Relative improvement coefficients of ELM-AE-R vs. PCR and PLSR.

	ELM-AE-R vs. PCR		ELM-AE-R vs. PLSR	
	Training	Testing	Training	Testing
RMSE	9.60%	18.54%	−19.67%	18.05%
R^2^	3.32%	8.12%	−8.15%	2.08%

**Table 4:** Results of evaluation metrics during regression analysis (11 PCs).

**Training Data**	**RMSE**			**R^2^**		
	**N_2_O**	**NO_2_**	**NO**	**N_2_O**	**NO_2_**	**NO**
PCR	0.7841	12.9753	18.1921	0.9965	0.9903	0.9844
PLSR	0.8372	7.95820	12.3824	0.9960	0.9963	0.9912
ELM-AE-R	0.8000	10.2871	13.3505	0.9964	0.9950	0.9916
**Testing Data**	**RMSE**			**R^2^**		
	**N_2_O**	**NO_2_**	**NO**	**N_2_O**	**NO_2_**	**NO**
PCR	0.8533	19.3015	59.1022	0.9986	0.9681	0.9081
PLSR	0.9415	31.0466	24.8107	0.9983	0.9176	0.9838
ELM-AE-R	0.9529	16.5519	23.1046	0.9983	0.9766	0.9860
